# Highly precise navigation at the lateral skull base by the combination of flat-panel volume CT and electromagnetic navigation

**DOI:** 10.1177/00368504211032090

**Published:** 2021-08-16

**Authors:** Johannes Taeger, Franz-Tassilo Müller-Graff, Tilmann Neun, Maria Köping, Philipp Schendzielorz, Rudolf Hagen, Kristen Rak

**Affiliations:** 1 Department of Otorhinolaryngology, Plastic, Aesthetic and Reconstructive Head and Neck Surgery, University Hospital Würzburg, Würzburg, Bavaria, Germany; 2 Institute for Diagnostical and Interventional Neuroradiology, University Hospital Würzburg, Würzburg, Bavaria, Germany

**Keywords:** Electromagnetic navigation, fpVCT, fiducial registration error, lateral skull base, otology, cochlear implantation

## Abstract

This study aimed to evaluate the feasibility and accuracy of electromagnetic navigation at the lateral skull base in combination with flat panel volume computed tomography (fpVCT) datasets. A mastoidectomy and a posterior tympanotomy were performed on 10 samples of fresh frozen temporal bones. For registration, four self-drilling titanium screws were applied as fiducial markers. Multi-slice computed tomography (MSCT; 600 µm), conventional flat panel volume computed tomography (fpVCT; 466 µm), micro-fpVCT (197 µm) and secondary reconstructed fpVCT (100 µM) scans were performed and data were loaded into the navigation system. The resulting fiducial registration error (FRE) was analysed, and control of the navigation accuracy was performed. The registration process was very quick and reliable with the screws as fiducials. Compared to using the MSCT data, the micro-fpVCT data led to significantly lower FRE values, whereas conventional fpVCT and secondary reconstructed fpVCT data had no advantage in terms of accuracy. For all imaging modalities, there was no relevant visual deviation when targeting defined anatomical points with a navigation probe. fpVCT data are very well suited for electromagnetic navigation at the lateral skull base. The use of titanium screws as fiducial markers turned out to be ideal for comparing different imaging methods. A further evaluation of this approach by a clinical trial is required.

## Introduction

Medical navigation in lateral skull base has been described for many years. It has proven helpful from the very beginning with regard to the safety of surgical interventions and can have a positive impact on the length of operations.^1^ However, registration and calibration have always been difficult. A recent survey of skull base surgeons revealed a moderate use of navigation systems in this area of surgery. The main reason for this was the insufficient accuracy of all systems.^2^

Different navigation systems have been used at the lateral skull base, like electromechanical systems,^3–5^ electromagnetic systems^6–12^ or optical systems.^1,7,13–18^

In some studies, the precision of these navigation modalities was specifically compared. Ecke et al.^7^ reported that different optical systems varied considerably in the accuracy between laboratory and clinic, whereas the electromagnetic system showed similar results in both settings.

For precise navigation, an exact registration is of major importance. Various approaches have been used for registration, like externally fixed systems including maxillary splint registration,^4,5,13^ fixed fiducial markers^9,12,14,17^ and combined techniques.^7,8,10^

In other studies, the different registration approaches were compared, for example, bone-implanted markers or contour-based laser surface registration with a newly developed maxillary dental splint device with a laterally mounted fiducial carrier. The registration accuracy was similar to the implanted screws and superior to surface registration.^15^

Different modalities of radiological imaging have been used for navigation, mainly multislice computed tomography (MSCT),^1,7–9,13–15,18^ but also flat panel computed tomography (fpCT)^17^ and flat panel volume computed tomography (fpVCT).^10^

A fpVCT system has an innovative design that allows higher spatial resolution for the visualization of complex human anatomy in comparison to MSCT scanners.^19,20^ In one study the resolution of a prototypic fpVCT scanner, a clinical available flat panel digital volume tomography (fpDVT) scanner and a conventional MSCT scanner for otologic procedures were compared. The authors described improvements of the image properties in temporal bone preparations for the newly developed methods.^21^ In a laboratory setting, the scalar position of cochlear implant electrodes could be precisely determined by an experimental fpVCT scanner.^22^ In a first investigation with a clinical available scanner for fpVCT a significantly higher overall image quality than MSCT was found. Furthermore, a reduction of the effective dose of approximately 40% was demonstrated for fpVCT compared to 46 section MSCT.^23^ Piergallini et al.^24^ compared the fpVCT with a 128 section MSCT and found higher image quality in temporal bone assessment because of the higher spatial resolution, with comparable equivalent doses but different effective doses to the organs. In addition, fpVCT also offers the possibility of enhancing image quality by a specially performed protocol (micro-fpVCT) to a resolution of <200 µm or by secondary reconstruction to a resolution of <100 µm.^25^

Taking these studies into consideration, the combination of fpVCT and electromagnetic navigation with registration by fiducial markers might provide a highly accurate system for navigation at the lateral skull base. FpVCT offers a high resolution down to 100 µm^25^ and a good suppression of metal artefacts, which is needed for good identification of the fiducial markers.^26^ Electromagnetic navigation has been shown to provide high accuracy at the lateral skull base.^12^ Therefore, the aim of the present study was to investigate whether the combination of fpVCT (466 µm), micro-fpVCT (197 µm) and secondary reconstructed fpVCT (fpVCT_RECO_; 98 µm) with an electromagnetic navigation system (Karl Storz NAV1^®^) can improve the accuracy of registration and navigation at the lateral skull base.

## Methods

### Specimens

For this study, 10 specimens of fresh frozen temporal bones were used. The temporal bone specimens were obtained following appropriate local guidelines and procedures for obtaining and using human tissue and the principles of the Declaration of Helsinki and Good Clinical Practice.

**Figure 1.**
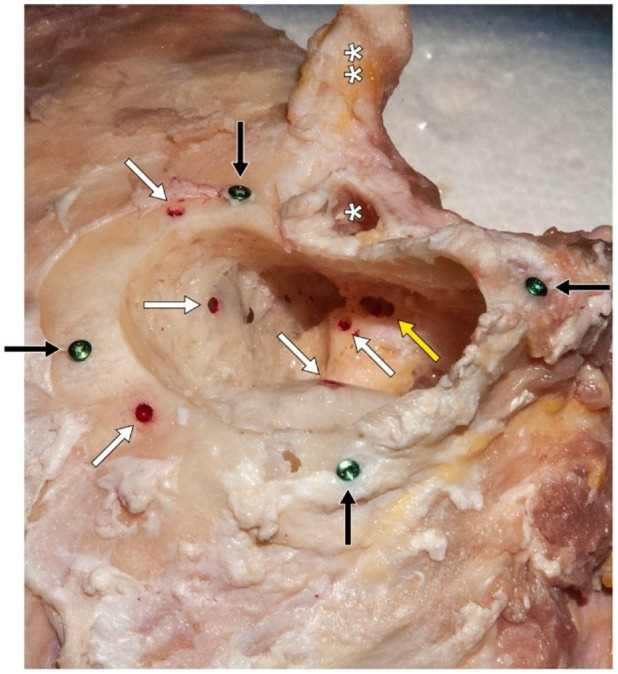
Photograph of a cadaveric temporal bone of the right side with titanium screws (black arrows) serving as registration markers. Drilled holes with red lacquer (white arrows) allow specific anatomical points to be reproducibly targeted with the navigation probe. The entrance to the round window niche, accessible via the posterior tympanotomy, was not marked with lacquer (yellow arrow). *Outer ear canal. **Zygomatic process.

Before preparation, the temporal bones were thawed at room temperature and then immersed in saline solution (0.9 %) for about 3 h. The experiments were done in a temperature-controlled laboratory (21 ± 1°C). A posterior tympanotomy was performed after a cortical mastoidectomy (Figure 1). Four self-drilling titanium screws of the Neuro Zti Implant set (kindly provided by Oticon GmbH, Hamburg, Germany) were inserted superiorly, anteriorly, posteriorly and inferiorly of the mastoidectomy as registration points. To repeatedly target specific structures on the temporal bone with the navigation probe as a criterion for the accuracy of the registration, several hollows with a diameter of 2 mm were bored with a diamond drill and marked with red lacquer. This was performed before the various CT scans were made in order to correlate these points visually accordingly with the imaging loaded into the navigation system. The following spots were marked by those hollows: temporal skullcap (anterior and posterior, each between two titanium screws), sigmoid sinus, dura and lateral semi-circular canal. The round window niche was also targeted with the probe, but not marked with lacquer.

The dissections were performed by an experienced otosurgeon (KR). The specimens appeared anatomically normal, as determined by light-microscopic examination.

### Imaging

Radiographic scans were performed using a fpVCT (Axiom Artis; Siemens Healthcare AG, Erlangen, Germany) and a MSCT system (SOMATOM Definition AS+; Siemens) with commercially available software (Syngo DynaCT; Siemens).

The fpVCT datasets were acquired using the following parameters: 20 s DCT Head protocol; tube current = 21 mA; tube voltage = 109 kV; rotation angle = 200°; pulse length = 3.5 ms; frame angulation step = 0.5°/frame; slice thickness = 466 µm. Secondary reconstructions (fpVCT_RECO_) from these data sets were performed with the following settings: 512 × 512 section matrix; HU kernel types; sharp image characteristics; slice thickness = 100 µm.

With the same hard- and software, micro-fpVCT was conducted using the following parameters: 20 s DCT Head protocol; tube current = 42 mA; tube voltage = 109 kV; rotation angle = 200°; pulse length = 3.5 ms; frame angulation step = 0.4°/frame; slice thickness: 197 µm.

The MSCT datasets were acquired using a SOMATOM Definition AS+ (Siemens) with commercially available software (Syngo CT; Siemens). The following parameters of the standard application (inner ear high-resolution program) were applied: tube current = 38 mA; tube voltage = 120 kV; collimation = 0.6 mm; pitch = 0.55; slice thickness = 600 µm.

### Electromagnetic navigation

**Figure 2.**
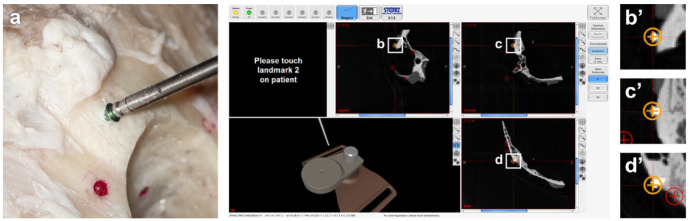
Usage of the inserted titanium screws as markers for electromagnetic navigation; photograph of the anatomical situs with the navigation probe on the head of a titanium screw (a), screenshot of the navigation software with display of the accurately shown contact of the probe with the screw head in sagittal (b), coronary (c) and axial (d) slices; corresponding magnifications (b’, c’, d’)

The NAV1^®^ electromagnetic navigation system (Karl Storz SE & Co. KG, Tuttlingen, Germany) was used according to the manufacturer’s instructions. The titanium screws served as fiducial markers in the software of the system. The registration points were placed manually with great care in all spatial levels in the place of the screw head, where the probe is most likely to touch down (Figure 2). After the registration process, the resulting *fiducial registration error* (FRE) values were noted. In addition, the various lacquer-marked hollows were touched with a probe in order to visually detect spatial deviations in the respective CT data set. No target registration error (TRE) was measured, since the NAV1^®^ electromagnetic navigation system allows only for calculating the TRE down to 1 mm.

### Statistical analysis

All statistical tests were selected before data collection. Statistical analyses were performed using Prism (version 8.2.1, GraphPad Software, La Jolla, CA, USA). A paired sample Student’s *t*-test was used to compare the mean of the fiducial registration error (FRE) of the different imaging methods. Data are reported as median and 95% confidence interval (CI). Differences with a *p*-value less than 0.05 were considered to be statistically significant. Data are presented in a graph as median ± standard deviation (SD) of the mean.

## Results

By using the titanium screws as markers, the registration process in the navigation software was very quick and reliable. This has proven to be a great advantage over the use of native anatomical structures, since the petrous bone offers few prominent points that can be easily correlated with the specimen in the radiologic images. In this experimental setup, the duration of importing the radiological data, setting the markers at the screw heads and registration was approximately 3 min. The fpVCT data could be easily integrated into the electromagnetic navigation system without any problems.

**Figure 3.**
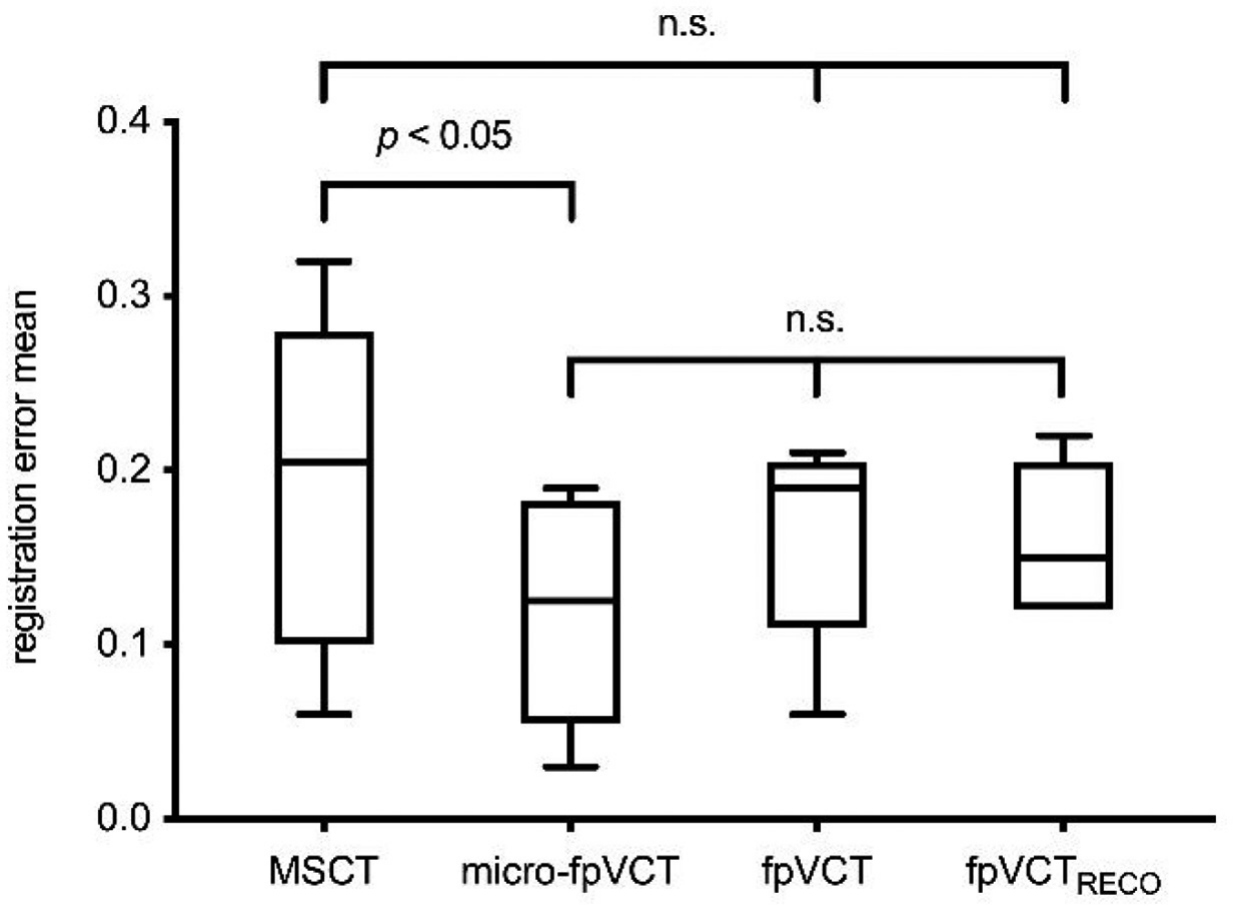
Box plots of the fiducial registration error mean for MSCT (600 µM), micro-fpVCT (197 µm), fpVCT (466 µm) and secondary reconstructions of the fpVCT data, fpVCT_RECO_ (RECO: reconstruction; 100 µM). Only MSCT and micro-fpVCT showed a significant difference. n.s.: not significant, bars: SD.

**Figure 4.**
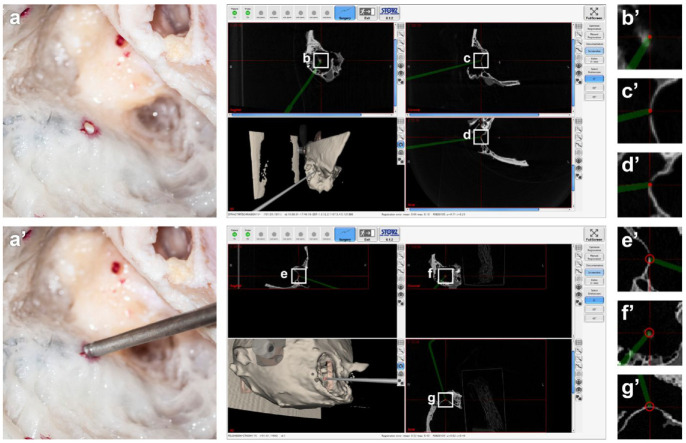
The coloured marking of the drilled hollows allows for reproducible targeting of specific anatomical structures in an experimental setting (a, a’; here: sigmoid sinus). The use of micro-fpVCT (top screenshot) resulted in significantly lower FRE values (represented by the size of the red circle at the tip of the probe, which is rendered by the software; b: sagittal, c: coronal, d: axial; corresponding magnifications: b’, c’; d’) compared to MSCT (lower screenshot; e: sagittal, f: coronal, g: axial; corresponding magnifications: e’, f’; g’). However, there was no visually detectable, clinically relevant deviation from the target structures with the MSCT data.

When comparing the different imaging methods, there was a significant difference in the FRE values only for MSCT (median: 0.21; 95% CI: 0.07–0.31) versus micro-fpVCT (median: 0.13; 95% CI: 0.04–0.19) data (Figure 3). The micro-fpVCT and the conventional fpVCT (median: 0.19; 95% CI: 0.06–0.21) data led to comparable results, with the micro-fpVCT tending to perform better (not significant [n.s.]). The use of the fpVCT_RECO_ (median: 0.15; 95% CI: 0.12–0.22) data also led to slightly lower REM values compared to the conventional fpVCT data (n.s.). Since the use of the titanium screws as reliable fiducial markers led to fairly good FRE values in all imaging modalities, there was no recognizable, clinically relevant deviation in the visual control of the target registration accuracy when targeting the hollows with the navigation probe (Figure 4).

## Discussion

Medical navigation systems enable real-time linking of an intraoperative site with imaging data. This allows the surgeon to continuously monitor the patient’s anatomy and the spatial position of surgical instruments. However, to find a broader application especially at the lateral skull base, the accuracy of these systems must be optimized.

One way to achieve this, as presented in this work, is to use imaging with high resolution like fpVCT. Compared to MSCT data (600 µm), it is characterized by a significantly higher resolution (466 µm). In order to further improve the image quality without additional radiation exposure, it is additionally possible to create secondary reconstructions with a resolution of <100 µm from fpVCT raw data^25^ or to perform a micro-fpVCT with primarily higher resolution of under 200 µm. Furthermore, the benefit of fpVCT is a 40% lower effective radiation exposure compared to 46-section and 128-section MSCT.^23,24^ Regarding the scanning time there is a difference between fpVCT and MSCT. In our study, the protocol used for the fpVCT scans required a time of 20 s. In contrast, a regular CT scan of the temporal bone takes between 4 and 5 s.As a result, the time for the fpVCT scan is around four to five times longer, which carries the risk of more movement artifacts in patients.

In several studies it was investigated if fpVCT can be used for navigation. Bartling et al.^27^ investigated whether using flat panel volume computed tomography (fpVCT) can improve the accuracy of navigation in a phantom model. The target accuracy could be significantly enhanced by the new imaging technique using an optical navigation system. Subsequently, the possible application of this combined system was shown in a cadaveric study with the purpose to investigate possible trajectories for robotic cochlear implant surgery.^28^

In the present study, the fiducial registration error (FRE) was measured after registration with fixed fiducial markers. The FRE is the root-mean square error in fiducial alignment between image space and physical space^29,30^ and is a criterion of the quality of registration. Although the FRE is not clearly correlated with the navigation accuracy, it shows if a registration can be used for clinical application. By the combination of fpVCT with electromagnetic registration, an FRE of under 0.5 mm could be achieved. In addition, a significantly lower FRE could be derived when using micro-fpVCT data compared to MSCT data. Conventional fpVCT data with a slice thickness of 466 µm and its secondary reconstructions (fpVCT with a slice thickness of >100 µm) tend to show lower FRE values, but these were not significantly different from MSCT. The disadvantage of the micro-fpVCT is the reduced volume of the scan, being only able to scan one temporal bone or in patients one side of the head, which would lead to double radiation exposure in a routine setting. But, if fiducial screws are applied for registration intraoperatively, an additional CT scan of the lateral skulls base is nevertheless needed. If a micro-fpVCT is performed instead, even a reduction of the radiation exposure is reached.

In medical navigation the target registration error (TRE) is in addition to the FRE a relevant factor for clinical applicability. The TRE specifies the accuracy with which a point in the imaging correlates with the patient’s anatomy.^29,31,32^ In the experiments performed in this study a TRE was not measured, since the NAV1^®^ electromagnetic navigation system allows only for calculating the TRE down to 1 mm and. A visible control revealed a fairly good concordance of the navigated points in the temporal bone specimens and the visualization of this point in the navigation system, which was – at least in our opinion – suitable for highly accurate navigation.

In other studies, electromagnetic navigation was also used at the lateral skull base. An extensive dissection of the temporal bone was performed in a recent investigation to test the accuracy of electromagnetic navigation with conventional MSCT scans. The TRE was about 0.5 mm. The authors state that electromagnetic navigation is sufficiently accurate to be used in a surgical setting.^12^ Bernardeschi et al.^9^ demonstrated that a TRE of under 1 mm can be obtained in a clinical setting using an electromagnetic navigation system. Up to now, in only one study an electromagnetic navigation in combination with a fpVCT was used, but the main interest of the study was to compare different registration protocols and not to test the different radiological examinations and secondary reconstructions of the fpVCT. The authors achieved a TRE at the mastoidal plane of 1.21 mm (10).

The portability of fpVCT is another relevant advantage as it enables intraoperative use, too. In combination with the lower metal artefacts,^26^ this is of crucial importance in respect of using titanium screws as markers that could be screwed in intraoperatively in the clinical setting.

The presented results emphasize that the use of titanium screws as artificial fiducial markers is essential for the comparison of different imaging modalities in the experimental setting. Since the petrous bone offers hardly any striking points that are sufficiently suitable for the registration process, an adequate comparison of the precision in the sub-millimetre range would otherwise not be possible. Furthermore, the procedure with drilled holes with lacquer marking in the petrous preparation has proven to be useful for reproducible visual control of various influencing variables during measurement.

One limitation of this work is that specimens were used. Taking into account that motion artefacts represent a significant factor influencing the image quality,^26,33^ this approach still has only experimental significance.

## Conclusion

The combination of fpVCT and electromagnetic navigation has the potential to improve the accuracy of navigation at the lateral skull base. This could be helpful in different clinical cases like tumours, excessive inflammatory disease, like cholesteatoma or aural atresia, but also in robot-assisted cochlear implantations. For the future, a fiducial based registration strategy with titanium screws combined with micro-fpVCT radiological data is proposed to optimize the registration of the patient and an electromagnetic system for highly accurate navigation at the lateral skull base. There is a need for a clinical study to clarify the possible advantages in a clinic setting.
